# Self-monitoring of blood pressure following a stroke or transient ischaemic attack (TASMIN5S): a randomised controlled trial

**DOI:** 10.1186/s12872-024-04320-0

**Published:** 2024-12-27

**Authors:** R. J. McManus, A. Smith, E. Temple, L. M. Yu, J. Allen, R. Doogue, G. A. Ford, L. Glynn, B. Guthrie, P. Hall, L. Hinton, F. D. R. Hobbs, J. Mant, B. McKinstry, G. Mead, K. Morton, T. Rai, C. Rice, C. Roman, A. Stoddart, L. Tarassenko, C. Velardo, M. Williams, L. Yardley, on behalf of the TASMIN5S investigators

**Affiliations:** 1 Nuffield Department of Primary Care Health Sciences, University of Oxford, Oxford, UK; 2 School of Medicine, University of Limerick, Limerick, Ireland; 3 Radcliffe Department of Medicine, University of Oxford, and Oxford University Hospitals NHS Foundation Trust, Oxford, UK; 4 Advanced Care Research Centre, Usher Institute, University of Edinburgh, Edinburgh, UK; 5 Department of Public Health and Primary Care Research, University of Cambridge, Cambridge, UK; 6 School of Psychology, University of Southampton, Southampton, UK; 7 Public contributor, Bristol, UK; 8 Public contributor, London, UK; 9 Institute of Biomedical Engineering, Department of Engineering Science, University of Oxford, Oxford, UK; 10 Edinburgh Clinical Trials Unit, Usher Institute, University of Edinburgh, Edinburgh, UK; 11 School of Psychological Science, University of Bristol, Bristol, UK; 12 Brighton and Sussex Medical School, Universities of Brighton and Sussex, Brighton, UK

**Keywords:** Hypertension, Blood pressure, Stroke, TIA, Self-monitoring

## Abstract

**Background:**

Blood pressure (BP) control following stroke is important but currently sub-optimal. This trial aimed to determine whether self-monitoring of hypertension with telemonitoring and a treatment escalation protocol, results in lower BP than usual care in people with previous stroke or transient ischaemic attack (TIA).

**Methods:**

Unblinded randomised controlled trial, comparing a BP telemonitoring-based intervention with control (usual care) for hypertension management in 12 primary care practices in England. People with previous stroke or TIA with clinic systolic BP 130–180 mmHg, taking ≤ 3 antihypertensive medications and on stable treatment for at least four weeks were randomised 1:1 using secure online system to intervention or control. The BP:Together intervention comprised self-monitoring of blood pressure with a digital behavioural intervention which supported telemonitoring of self-monitored BP with feedback to clinicians and patients regarding medication titration. The planned primary outcome was difference in clinic measured systolic BP 12 months from randomisation but was not available following early study termination due to withdrawal of funding during the COVID-19 pandemic. Instead, in addition to pre-randomised data, routinely recorded BP was extracted from electronic patient records both pre- and post-randomisation and presented descriptively only. An intention to treat approach was taken.

**Results:**

From 650 postal invitations, 129 (20%) responded, of whom 95 people had been screened for eligibility prior to the pandemic (November 2019-March 2020) and 55 (58%) were randomised. Pre-randomisation routinely recorded mean BP was 145/78 mmHg in the control (*n* = 26) and 145/79 mmHg in the self-monitoring (*n* = 21) groups. Post-randomisation mean BP was 134/73 mmHg in the control (*n* = 19) and 130/75 mmHg in the self-monitoring (*n* = 25) groups. Participants randomised to self-monitoring used the intervention for ≥ 7 months in 25/27 (93%) of cases.

**Conclusions:**

Recruitment of people with stroke/TIA to a trial comparing a BP self-monitoring and digital behavioural intervention to usual care was feasible prior to the COVID-19 pandemic and the vast majority of those randomised to intervention used it while the trial was running. Routinely recorded blood pressure control improved in both groups. Digital interventions including self-monitoring are feasible for people with stroke/TIA and should be definitively evaluated in future trials.

**Trial registration:**

ISRCTN57946500 06/09/2019 Prospective.

**Supplementary Information:**

The online version contains supplementary material available at 10.1186/s12872-024-04320-0.

## Introduction

High blood pressure is the most important treatable modifiable risk factor for recurrent stroke, with relative risk increasing by about one third for every 10 mmHg increase in systolic blood pressure (BP) [1]. People who have survived previous stroke or transient ischaemic attack (TIA) are at particularly high risk of subsequent stroke, but often have sub-optimal BP control with many remaining above target levels recommended in guidelines [2–4]. Furthermore, people with stroke/TIA may have ongoing communication and mobility deficits which affect their ability to access care [2]. Potentially modifiable reasons for poor BP control post stroke include clinical inertia, poor adherence to medication, organisational failure and lack of engagement of carers and these should all be targeted by novel interventions [5–8].

Previous work by this group has shown that General Practitioner (GP) supervised self-monitoring and self-management of hypertension are effective at lowering blood pressure in primary care through optimising prescription of antihypertensives and providing a structure of care for clinicians and patients alike [9–11]. Mobile phones are now commonplace for all age groups and can provide a platform for novel interventions [12, 13]. However, few data exist in patients with stroke/TIA and those that do are largely nurse-led telephone based interventions without systematic telemonitoring of blood pressure [14, 15].

The original aim of the TASMIN5S trial was to determine whether an integrated intervention (BP:Together) developed with people with previous stroke/TIA using a person-based approach [16], combining self-monitoring/management of hypertension with telemonitoring and a treatment escalation protocol, resulted in lower blood pressure than usual care in people with previous stroke or TIA. However, the trial was paused due to the COVID-19 pandemic, and funding withdrawn by the funder in November 2020 rather than allowing continuation. This paper reports on the initial recruitment and baseline data collected November-March 2020, along with intervention usage and routine data from randomised individuals to assess the feasibility of the trial intervention as it is important to publish available trial data to inform future research [17].

## Methods

This was an unblinded, multicentre, primary care based, individually randomised controlled trial.

### Participants

People with a recorded history of stroke or transient ischaemic attack (TIA, at least four week prior to recruitment), aged ≥ 18 years, with clinic systolic blood pressure 130–180 mmHg [18], taking ≤ 3 antihypertensive medications and without changes to antihypertensive therapy for at least four weeks, were invited to participate. Exclusion criteria were orthostatic hypotension (≥ 20mmHg systolic drop), pregnancy, atrial fibrillation (due to lack of digital monitor validation) [19], dementia, a score over 10 in short orientation memory concentration test (unless able to consent with willing carer) [20], stage 4/5 chronic kidney disease or with proteinuria (these might affect care pathways) [21], diastolic BP > 110mmHg, participation in another hypertension trial, unwilling to self-monitor or receiving specialist hypertension care.

### Procedures

Potentially eligible participants were identified using automated searches of electronic primary care patient records and invited by post to take part using an aphasia-friendly covering letter and information booklet giving study details [16]. At the baseline clinic, completed in the practice by research staff (practice base or research team), written informed consent was obtained before eligibility confirmation including BP measurement using a validated monitor [22]. Before randomisation, patients completed baseline questionnaires and past medical history and current medication were recorded from the clinical record.

Participants were randomised (1:1) to either usual care or the BP:Together intervention [16], minimised by modified Rankin Scale (0/1 vs 2–5 [there is a typo in the protocol which reads 2–4]) and baseline blood pressure (above/below 145 mmHg systolic) using a secure, automated online system.

All participants attended a baseline medication review with a GP or other prescriber after randomisation. The clinician made changes to their antihypertensive medication if indicated and choice of antihypertensive medication was at the prescriber’s discretion regardless of randomisation group. All participating practices received recommendations for appropriate secondary prevention based on then then current National Clinical Guidelines [18].

The trial was prospectively registered: ISRCTN 57946500 (06/09/2019).

#### Usual care

Patients in the control group received usual hypertension care as recommended by their practice with no specified monitoring or medication regime.

#### BP: Together intervention

Participants in the intervention group were trained by the research nurse to take their own BP and to use the BP: Together intervention [16]. This was specifically developed for the trial with extensive input from patient and public involvement (PPI) contributors (two trial team members with lived experience of stroke), as well as research participants who were either stroke patients and/or their carers, and primary care health professionals attending stroke patients. The intervention included stroke specific BP targets and participant options for the use of a smartphone app, a secure website or an SMS/text-based service for telemonitoring [Table 1]. Participant training (approximately 30 min) was supported by video instructions and support booklets designed to be accessible to stroke/TIA survivors (including those with aphasia or physical disability) and to increase confidence and motivation for self-monitoring (for example, by explaining the rationale for lowering BP after a stroke).

**Table 1:** BP:Together intervention components

Participant	Clinician
BP monitor (Omron M10)	Monthly reports based on patient data
App/website/SMS interface comprising	Website including
Electronic patient training booklet^a^	BP readings history including real time graphical interface
Record of past BP readings including graphical interface^a^	Direct messaging to participants (one way)
Reminders & Feedback	Clinician training resources
Data entry	

^a^not available on SMS interface; manual version available

Participants were asked to self-monitor their BP twice each morning and each evening for at least 3 days every 4 weeks (minimum of 12 measurements/4 weeks, further referred as a ‘monitoring period’) using validated monitors (supplied by the trial) and to transmit readings using the BP:Together digital intervention (ie via app, website or text message). An additional 12 readings were requested if blood pressure was close to threshold (mean systolic 140mmHg ± 5mmHg).

A home target systolic blood pressure of 125 mmHg was chosen to be consistent with the 130 mmHg clinic target then recommended by the National Clinical Guidelines for Stroke [18]. GPs received patient specific summaries of self-monitored BP by email each month [16].

For participants in the intervention group, at the initial medication review, the prescriber made a plan for 3 potential medication changes to be made if average self-monitored BP was raised. Intervention group participants received automated prompts to act on this plan in the light of their BP. Safety limits allowed prompt clinical review in the presence of very high or very low readings. Both participants and GPs used a previously developed traffic light system to guide medication titration [16].

The original trial plan is outlined in the flow chart in appendix 1.

#### Outcomes

The primary outcome was difference in clinic measured systolic blood pressure, adjusted for baseline and other covariates at 12 months*.*Secondary outcomes included medication adherence (MARS) [23], beliefs about medicines (BMQ) [23] quality of life (EQ-5D 5L) [24], anxiety (STAI-6 item) [25], patient enablement [26, 27] and lifestyle questions regarding smoking and alcohol and a modified Rankin Scale (mRS, a 7 point scale designed to assess disability after stroke ranging from 0 (no symptoms) to 5 (severe disability) and 6 (dead)) [28]. The trial was stopped early due to the pandemic and so the planned primary outcome could not be collected. In order to gain as much information as possible from those randomised in the trial, data concerning blood pressure, medications and consultations were extracted from the clinical record for the period comprising one year prior to all randomisation (1/NOV/2018) until the last day of the study (1/DEC/2020). Usage data were collected from the BP:Together intervention for each four week monitoring period during the trial.

#### Sample size

A sample size of 244 people per group was required for 90% power assuming a standard deviation of 17 mmHg to detect a difference of at least 5 mmHg between intervention and control groups. Assuming a 20% drop out (including death, withdrawal, or moving away), a sample of 305 per group was needed; a total of 610 participants.

#### Analysis

As less than 20% of patients were recruited before the trial was terminated, all analyses were descriptive in nature and do not include comparisons or 95% confidence intervals. Baseline data and routinely collected clinical data pre- and post-randomisation are presented separately. The analysis utilised pre-randomisation data from 1 year prior to randomisation and post-randomisation data for 1 year after randomisation or up to and including 01/DEC/20 (whichever came first). For blood pressure for example, this means that all available BP data pre and post randomisation for each patient was used to arrive at the means which are presented without statistical comparison. The original statistical analysis plan and subsequently revised plan are presented in the supplementary material.

## Results

Participants were recruited between November 2019 and March 2020 when the study was paused due to COVID-19 pandemic restrictions. The trial design was urgently revised to allow remote recruitment, however funding was withdrawn by the funder due to the impact of COVID-19 on their available resources and priorities. The reasons given for withdrawal of funding were a) changes in practice due to the pandemic affecting the control arm rates of self-monitoring and subsequent impact on study power, b) concerns that primary care practices would not be able to engage in research due to covid and c) that fully remote recruitment would be challenging and could lead to selection bias.

**Fig. 1:**
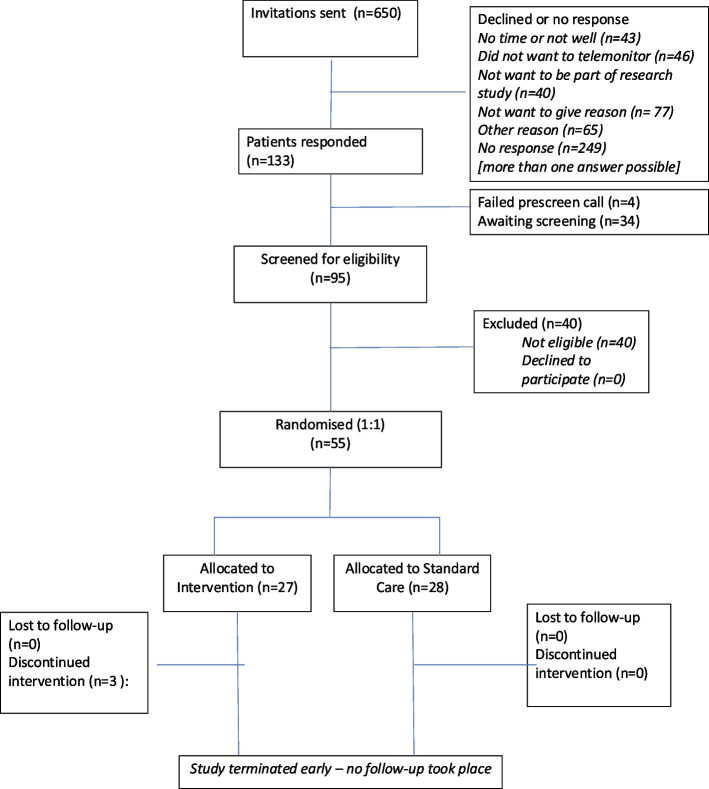
Flow through the trial

From the first seventeen practices participating (total practice population 236,562), 774 (0.4%) patients had been identified from electronic searches and GP list review to be invited. Of these, twelve practices with an invitation population of 650 patients were able to hold at least one recruitment clinic by the point of recruitment cessation and are included for further consideration of feasibility [Fig. 1]: 129 (20%) patients had responded to an invitation and passed prescreening. Of these, 95 (74%) had been seen at a baseline clinic and of these 55 (58%) recruited with 40 (42%) excluded on the basis of eligibility. A further 34 patients were waiting eligibility screening.

Of those screened and ineligible, 34/40 (85%) had blood pressure already controlled < 130 mmHg systolic, four were unwilling or unable to undertake trial procedures and two each were found to have very high blood pressure requiring urgent intervention (> 180/110 mmHg), or be taking more than three antihypertensives, or to have a significant postural drop (appendix 2 table S1 (all supplementary tables are included in appendix 2). The remainder of those prescreened (34 patients) were waiting for a baseline clinic when recruitment was halted.

In terms of overall feasibility, if the response (20%) and recruitment (58%) rates seen in the initial 12 practices were extrapolated to the planned sample size of 610, the trial would have required approximately a further 87 practices (99 total) which was in line with the predictions made in the funding application (100 practices).

Of 55 people randomised, the mean age was 74 years, 44% were female, 98% were of white ethnicity, 21 (38%) had previously had a stroke and 34 (62%) a TIA [Tables 2 and S2]. Baseline BP was 147/82 mmHg (27 participants, intervention group) and 146/80 mmHg (28 participants, control group) and participants were taking a mean of 0.9 and 1.2 antihypertensive medications respectively which related to a mean of 1.9 and 1.5 defined daily doses [Tables 2, S3 and S4]. The median modified Rankin scale was below one in both groups [23]. Three (11%) patients in the intervention group discontinued the intervention before the trial was stopped. Results are reported on an intention to treat basis.

**Table 2:** Baseline characteristics by randomised group

Characteristic (no missing data unless stated)	Self-monitoring (*N* = 27)	Control (*N* = 28)
**Age**		
Mean (SD)	72.2 (8.2)	76.1 (7.2)
**Gender**		
Female, n (%)	14 (51.9%)	10 (35.7%)
**Social environment**		
Live alone	6 (22.2%)	7 (25.0%)
Live with carer	1 (3.7%)	2 (7.1%)
**Ethnicity**		
White British, n (%)	26 (96.3%)	28 (100.0%)
Indian, n (%)	1 (3.7%)	0
**Level of education**		
University-undergraduate degree or higher, n (%)	9(33.3%)	9 (32.1%)
Secondary-school/higher certificate or GCE O/A-level, n (%)	12 (44.4%)	10 (35.7%)
Other qualification, n (%)	6 (22.2%)	8 (28.6%)
No qualifications, n (%)	0	1 (3.6%)
**Systolic blood pressure (mean of 2nd and 3rd reading) (mmHg) recorded at baseline clinic**		
Mean (SD)	146.5 (8.7)	145.9 (11.1)
**Diastolic blood pressure (mean of 2nd and 3rd reading) (mmHg) recorded at baseline clinic**		
Mean (SD)	81.9 (9.4)	80.3 (7.7)
**Current antihypertensive medication (number of)**		
Mean (SD)	0.9 (0.8)	1.2 (0.6)
**Modified Rankin scale**		
Not/mildly impaired (0–1)	22 (81.5%)	23 (82.1%)
Impaired (2–5)	5 (18.5%)	5 (17.9%)
[Range]	[0 to 3]	[0 to 3]
**Smoker**		
Yes, n (%)	2 (7.4%)	1 (3.6%)
**Past Cardiovascular History**		
Stroke, n(%)	12 (44.4%)	9 (32.1%)
TIA, n(%)	15 (55.6%)	19 (67.9%)
Myocardial infarction (heart attack), n (%)	1 (3.7%)	1 (3.6%)
Angina, n (%)	2 (7.4%)	0
Peripheral vascular disease, n (%)	1 (3.7%)	2 (7.1%)
Diabetes-Type II, n (%)	2 (7.4%)	3 (10.7%)
Heart failure, n (%)	1 (3.7%)	0
CABG/angioplasty/stent, n (%)	3 (11.1%)	2 (7.1%)

Data regarding routinely recorded BP during the study were available from 47/55 (85.5%) pre-randomisation and 44/55 (80%) post-randomisation) with a median follow-up time of 291 days (intervention 291, control 289 days). Considering all available data, at baseline, mean BP was 145/79 mmHg (intervention) and 145/78 (control) respectively [Table 3]. Following randomisation mean BP was 130/75 mmHg in the intervention group and 134/73 mmHg in the control group. Including only individuals with at least one BP in both periods made no material difference to the results (Table S3).

**Table 3:** Mean blood pressure measurements from routine clinical notes, pre-randomisation and post-randomisation^a^

	**Intervention**		**Control**	
	**Pre-randomisation (** ***N*** ** = 21)**	**Post-randomisation (** ***N*** ** = 25)**	**Pre-randomisation (** ***N*** ** = 26)**	**Post-randomisation (** ***N*** ** = 19)**
**Systolic blood pressure (mmHg)**				
Mean (SD)	144.8 (15.5)	129.7 (9.5)	144.8 (9.6)	134.0 (9.9)
Median (IQR)	140.0 (137.3 to 152.7)	130.7 (122.1 to 135.0)	144.0 (138.0 to 149.0)	133.0 (129.0 to 140.0)
[Range]^b^	[116.2 to 188.0]	[111.0 to 151.0]	[129.0 to 170.0]	[110.0 to 152.5]
Missing, n (%)	6 (22.2%)	2 (7.4%)	2 (7.1%)	9 (32.1%)
**Diastolic blood pressure (mmHg)**				
Mean (SD)	79.3 (10.1)	74.8 (8.0)	78.3 (7.5)	73.2 (6.1)
Median (IQR)	81.0 (74.3 to 84.0)	74.6 (70.8 to 80.0)	78.5 (74.7 to 83.5)	72.0 (68.0 to 78.3)
[Range]^b^	[60.3 to 100.0]	[60.0 to 95.0]	[54.5 to 92.0]	[62.0 to 84.0]
Missing, n (%)	6 (22.2%)	2 (7.4%)	2 (7.1%)	9 (32.1%)

^a^The data presented here are pre-randomisation clinical data from 1 year prior to randomisation and post-randomisation clinical data for 1 year after randomisation or up to and including 01/12/20 (whichever came first)

^b^Because these are routinely collected data as opposed to trial recorded data, baseline blood pressure presented here was not necessarily in line with the inclusion criteria. See Table 1 for baseline blood pressure data measured during trial inclusion

Of the 27 people randomised to the intervention group, 15 used text messages to transmit BP readings, 8 the web interface and 4 the app. The total number of BP readings submitted from all participants was 3510 with an average of 130 BP readings per participant, IQR [123, 152]. There were 27 BP readings that were submitted as confirmation of very high (Systolic > 180 mmHg, or Diastolic > 100 mmHg) or very low blood pressures (Systolic < 101 mmHg).

The 3510 BP readings were completed in 246 monitoring periods with a median of 12 blood pressure readings submitted per monitoring period, IQR [12, 12] Range [2, 14]. In 51 cases the monitoring period was extended by another 12 readings due to being near the 135mmHg systolic threshold initially.

Participants used the system for a median 9 monitoring periods, IQR [10, 11] reflecting the median follow-up of 291 days (approximately 9½ months). All 27 participants used the system for more than one monitoring period (4 weeks). Participants monitored on average for 86.7% of the available monitoring periods, IQR [90.1, 100] (median of 100%). 25 (93%) participants used BP: Together for 7 or more monitoring periods (≥ 7 months).

Routinely collected medication data were available from all participants (55, 100%). People in the intervention group were prescribed any antihypertensive in 18/27 (66.7%) pre- and 22/27 (81.5%) of cases post-randomisation. Those in the control group were prescribed antihypertensives in 25/28 (89.3%) and 21/28 (75%) of cases pre- and post-randomisation respectively [Table 4]. The median number of antihypertensives was 1.0 in both groups pre- and post-randomisation.

**Table 4:** Antihypertensive medications from TASMIN5S trial data pre-randomisation and routine clinical notes post-randomisation^a^

	**Intervention**		**Control**	
	**Pre-randomisation (** ***N*** ** = 27)**	**Post-randomisation (** ***N*** ** = 27)**	**Pre-randomisation (** ***N*** ** = 28)**	**Post-randomisation (** ***N*** ** = 28)**
Any antihypertensives taken, n (%)	18 (66.7%)	22 (81.5%)	25 (89.3%)	21 (75.0%)
Mean (SD)	0.9 (0.8)	1.2 (0.8)	1.2 (0.6)	1.0 (0.7)
Median (IQR)	1.0 (0.0 to 2.0)	1.0 (1.0 to 2.0)	1.0 (1.0 to 2.0)	1.0 (0.5 to 1.5)
[Range]	[0.0 to 2.0]	[0.0 to 3.0]	[0.0 to 2.0]	[0.0 to 2.0]

^a^The data presented comprise pre-randomisation trial data and post-randomisation clinical data for 1 year after randomisation or up to and including 01/12/20 (whichever came first)

Pre-randomisation, 16/27 (59.3%, intervention) participants and 15/28 (53.6%, control) had at least one hypertension related consultation. Following randomisation, the equivalent proportions were 26/27 (96.3%, intervention) and 28/28 (100%, usual care) respectively [Table S5]. Consultation rates were a median of 1 in both groups pre randomisation and post randomisation 3 (interquartile range 2, 5) (intervention) and 1.0 (IQR 1, 2) (control) [Table S5]. The majority of recorded consultations were with GPs rather than nurses in both groups with an apparent increase in the proportion with GP consultations post randomisation.

Three serious adverse events were reported, all in the intervention group [Table S6]. None were considered to be related to the intervention.

## Discussion

### Main findings

This primary care-based trial of a self-management intervention was terminated early due to the withdrawal of funding in response to the COVID-19 pandemic, thus precluding definitive conclusions. Routinely collected data showed that blood pressure improved in both groups but the sample size was too small to draw conclusions about efficacy.

This was planned to be the first trial of self-management following TIA/stroke with a digital intervention specifically designed for people with stroke. The main limitations were the insufficient sample size and an inability to collect the planned primary outcome due to early termination. Although the trial protocol had undergone wholesale revision to reorientate to remote methods due to the pandemic, further recruitment and follow-up were not possible following withdrawal of funding. Consequently, no data are available as to whether the intervention would have reduced blood pressure more than usual care, as has been seen in other studies [14].

The use of routinely collected data for follow-up, while planned prospectively for the economic analyses, cannot adequately replace planned follow-up measurements and is likely to have been biased by the impact of the pandemic. Routinely collected blood pressure data are not the same as carefully measured trial outcomes but were all that was available in the circumstances [29]. This was an unblinded trial and so BP measurements were likely taken with knowledge of intervention allocation. In terms of workload, economic evaluation methods for new interventions in the United Kingdom inherently require comparisons of cost-efficiency to be relative to usual care [30]. UK primary care underwent very significant changes in consulting patterns in March/April 2020 and these have subsequently been shown to have reduced blood pressure measurement in primary care [31]. This will have impacted the control group especially as “usual care” was far from pre-pandemic norms. The possible differences in consulting rates may therefore have been simply due to those in the intervention group having self-monitoring data to discuss remotely with their GPs in comparison to the control group with reduced clinic blood pressure monitoring. Similar initial increases in consulting associated with medication titration were seen in our previous HITS trial [9]. Furthermore, the need to revert intervention group patients to usual care following trial cessation may have increased apparent consultation rates compared to those already receiving usual care. Future trials will need to understand the “new normal” following subsequent implementation of the BP@Home initiative in England from 2021 (comprising the distribution of self-monitoring equipment and implementation of remote BP monitoring for high-risk groups by NHS England which started after trial cessation, https://nhshealthcall.co.uk/product/bp-at-home/), in order to establish an appropriate comparator.

With these caveats, routinely collected data suggest that BP reduced in both randomisation groups, similar to our previous trials [32, 33]. This is likely driven by regression to the mean which is an issue in all trials where inclusion criteria include blood pressure above target [34], and which also affects evaluation of implementation of self-monitoring in routine practice unless a control group is used. The sample size achieved was not sufficient to determine if there was a true difference between groups due to the intervention, which could only be evaluated in a definitive trial.

The initial phases of the trial were sufficient to draw some conclusions regarding feasibility. Prior to suspension of the trial, 55 people with stroke had been recruited from 12 practices with an almost 60% conversion rate from screening, and further people waiting to be screened at the point of trial suspension. Furthermore, the vast majority of those randomised to self-management successfully used the BP:Together intervention. The high compliance in completing monitoring periods indicate high engagement with the digital intervention and its design. This suggests feasibility and acceptability under normal circumstances and is similar to other work by our group following stroke [35]. People randomised to the study were on average in their mid-70s, relatively fit, and did not live with a carer, so the initial cohort randomised did not provide insight into whether the trial would be suitable for a more disabled stroke population who might conceivably benefit more from a remote care model and for whom the intervention was designed. Time will tell what future care pathways will emerge but more remote care seems likely. Indeed the most recent national stroke guideline in the UK recommends ambulatory and self-monitoring to monitor long term care [2]. Understanding patient preferences for communication via text messages, an app or web interface should be an important component of future investigation.

Self-monitoring of BP is not currently appropriate for those with atrial fibrillation (AF) due to the lack of validated home monitoring equipment [19]. This reduces generalisability in stroke, although emerging data from ambulatory monitoring suggests that the increased number of measurements used in home monitoring might also reduce inaccuracies otherwise inherent in measurement in atrial fibrillation [36]. Most participants were white British reflecting both the national ethnicity mix in higher age strata, and that recruitment was largely confined to Oxfordshire in the initial phases of the study. Future work should ensure that ethnic minorities are well represented in recruited populations [37]. In comparison to some other self-monitoring studies, participants were largely educated to pre-university level suggesting the intervention may have been widely applicable as designed [14].

The decision to stop the trial was taken by the funders in a background of reduced income and much uncertainty [38]. Whilst around 40% of people with hypertension self-monitor in the UK, many do not tell their GP and there are no data on self-monitoring following stroke, where blood pressure is an important risk factor [39]. As we write in 2024, there remains a need to trial a telemonitoring intervention designed to be used by people following stroke and TIA and it is unfortunate that this study has not been able to provide this.

## Conclusions

Recruitment to a trial of a digital intervention designed to improve BP control following TIA/Stroke was feasible until suspended due to the COVID-19 pandemic. The majority of those allocated to the intervention used it as long as it was available. People randomised to either group showed improvement in their recorded BP but insufficient recruitment occurred for formal assessment of the efficacy of a digital tool to enable BP self-management. Understanding the most appropriate and effective roles for digital behavioural interventions in the management of people with poorly controlled blood pressure and a history of stroke or TIA requires further research.

## Supplementary Information

Supplementary Material 1.

## Data Availability

Data are available from the authors upon reasonable request and with permission of the University of Oxford. Use of data will require approval by an independent review committee identified for this purpose and investigators requesting data will need to provide a written protocol including analysis plan and sign a data sharing/access agreement. Requests for Data Sharing should be directed to information.guardian@phc.ox.ac.uk.

## Abbreviations

BP: Blood Pressure
GP: General Practitioner
mmHg: Millimetres of Mercury
NHS: National Health Service
NIHR: National Institute for Health Research
SMBP: Self-monitoring of blood pressure
TIA: Transient Ischaemic Attack
