# Supplementary material for: Self-monitoring of blood pressure following a stroke or transient ischaemic attack (TASMIN5S): a randomised controlled trial
Source: BMC Cardiovasc Disord. 2024 Dec 27;24:746. doi: 10.1186/s12872-024-04320-0 (PMC11673707; doi:10.1186/s12872-024-04320-0)
Supplement: Supplementary file 1 — Supplementary Material 1. [file 12872_2024_4320_MOESM1_ESM.docx]

**Web Appendices**

Appendix 1 Flow chart for study as originally envisaged

Appendix 2 Additional tables

**Appendix 1 Planned Study Flow Chart**


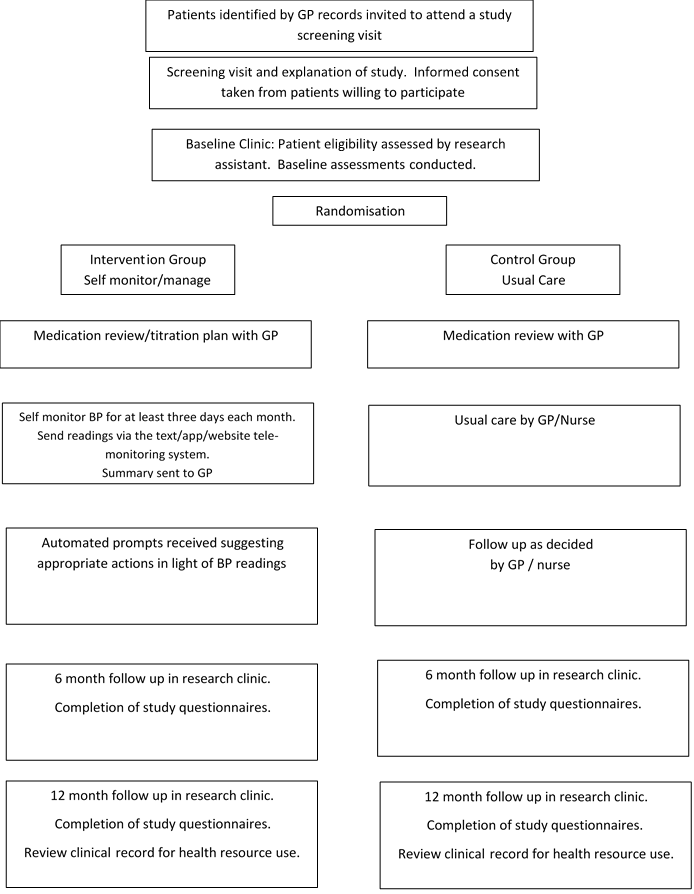


**Appendix 2 Additional tables**

Table S1 Reasons for ineligibility at baseline

| **Reason found ineligible at baseline** | **Frequency** |
| --- | --- |
| Baseline systolic blood pressure ≤ 130mmHg (mean of 2nd and 3rd readings) | 34 |
| No previous history of stroke and/or specialist confirmed TIA at least one month before randomisation | 1 |
| Not willing and able to comply with all study procedures either independently or with help from carer | 4 |
| Score over 10 on the short orientation memory concentration test | 1 |
| BP >180/110 mmHg at baseline | 2 |
| Currently taking more than three antihypertensive medicines | 2 |
| Receiving care for blood pressure by a specialist rather than a primary care physician | 1 |
| Stage 4 CKD or worse – i.e.: eGFR less than 30 mL/min/1.73 m2 | 1 |
| Diagnosed atrial fibrillation | 1 |
| Orthostatic hypotension: more than 20mmHg systolic drop after standing for 1 min | 2 |
| **Total no. of participants ineligible at baseline visit*:** | **40** |

*Some participants were ineligible for more than one eligibility criterion

**Additional baseline data including additional patient reported measures**

Table S2 additional Baseline characteristics by randomised group

| **Characteristic** | **Intervention (N=27)** | **Control (N=28)** | | | **Overall (N=55)** |
| --- | --- | --- | --- | --- | --- |
| **Cuff size for clinical blood pressure readings** |  |  | | |  |
| Small, n (%) | 1 (3.7%) | 1 (3.6%) | | | 2 (3.6%) |
| Regular, n (%) | 22 (81.5%) | 24 (85.7%) | | | 46 (83.6%) |
| Large, n (%) | 4 (14.8%) | 3 (10.7%) | | | 7 (12.7%) |
| **Arm used for clinical blood pressure readings** |  |  | | |  |
| Left, n (%) | 27 (100.0%) | 27 (96.4%) | | | 54 (98.2%) |
| Right, n (%) | 0 | 1 (3.6%) | | | 1 (1.8%) |
| **Short orientation memory concentration test score*^19^** |  |  | | |  |
| Median (IQR) | 0.0 (0.0 to 2.0) | 0.0 (0.0 to 2.0) | | | 0.0 (0.0 to 2.0) |
| [Range] | [0.0 to 10.0] | [0.0 to 10.0] | | | [0.0 to 10.0] |
| **Medication adherence rating scale** |  | |  |  | |
| Median (IQR) | 25.0 (24.0 to 25.0) | | 24.0 (23.0 to 25.0) | 25.0 (24.0 to 25.0) | |
| [Range] | [22.0 to 25.0] | | [13.0 to 25.0] | [13.0 to 25.0] | |
| *Missing, n (%)* | *6 (22.2%)** | | *1 (3.6%)** | *7 (12.7%)** | |

Error scores of 0-6 considered normal ^19^

| **Beliefs about medicines questionnaire (necessity subscale)** |  |  |  |
| --- | --- | --- | --- |
| Mean (SD) | 16.0 (4.1) | 16.1 (3.3) | 16.1 (3.6) |
| [Range] | [6.0 to 22.0] | [8.0 to 24.0] | [6.0 to 24.0] |
| *Missing, n (%)* | *6 (22.2%)** | *1 (3.6%)** | *7 (12.7%)** |
| **Beliefs about medicines questionnaire (concerns subscale)** |  |  |  |
| Mean (SD) | 9.8 (2.7) | 11.0 (3.3) | 10.5 (3.1) |
| [Range] | [5.0 to 14.0] | [5.0 to 19.0] | [5.0 to 19.0] |
| *Missing, n (%)* | *6 (22.2%)** | *1 (3.6%)** | *7 (12.7%)** |
| **Beliefs about medicines questionnaire (necessity-concerns differential)** |  |  |  |
| Mean (SD) | 6.2 (4.6) | 5.1 (3.8) | 5.6 (4.2) |
| [Range] | [-1.0 to 15.0] | [-7.0 to 14.0] | [-7.0 to 15.0] |
| *Missing, n (%)** | *6 (22.2%)* | *1 (3.6%)* | *7 (12.7%)* |
| **Six-item state-trait anxiety inventory (scaled)** |  |  |  |
| Mean (SD) | 28.5 (9.7) | 27.5 (8.1) | 28.0 (8.8) |
| [Range] | [20.0 to 50.0] | [20.0 to 46.7] | [20.0 to 50.0] |
| *Missing, n (%)* | *0* | *0* | *0* |

*The Beliefs about Medicines questionnaire and Medication Adherence Scale questionnaire could only be completed by participants who were taking antihypertensive medications. The 7 participants who did not answer any of these questions were not taking antihypertensive medications at baseline, therefore these questionnaires were not applicable for them

| **Characteristic**  **(no missing data unless stated)** | **Self-monitoring (N=27)** | **Usual care (N=28)** | **Overall (N=55)** |
| --- | --- | --- | --- |
| **Symptoms** |  |  |  |
| Stiff joints, n (%) | 13 (48.1%) | 17 (60.7%) | 30 (54.5%) |
| Fatigue, n (%) | 17 (63.0%) | 12 (42.9%) | 29 (52.7%) |
| Pain, n (%) | 16 (59.3%) | 12 (42.9%) | 28 (50.9%) |
| Cough, n (%) | 15 (55.6%) | 12 (42.9%) | 27 (49.1%) |
| Swelling of legs/ankles, n (%) | 12 (44.4%) | 9 (32.1%) | 21 (38.2%) |
| Loss of libido, n (%) | 12 (44.4%) | 7 (25.0%) | 19 (34.5%) |
| *Missing, n (%)* | *2 (7.4%)* | *1 (3.6%)* | *3 (5.5%)* |
| Loss of strength, n (%) | 10 (37.0%) | 8 (28.6%) | 18 (32.7%) |
| Pins and needles, n (%) | 10 (37.0%) | 8 (28.6%) | 18 (32.7%) |
| Dry mouth, n (%) | 9 (33.3%) | 9 (32.1%) | 18 (32.7%) |
| Mood change, n (%) | 7 (25.9%) | 10 (35.7%) | 17 (30.9%) |
| Breathlessness, n (%) | 7 (25.9%) | 10 (35.7%) | 17 (30.9%) |
| Sleep difficulties, n (%) | 10 (37.0%) | 7 (25.0%) | 17 (30.9%) |
| Headaches, n (%) | 8 (29.6%) | 8 (28.6%) | 16 (29.1%) |
| Wheeziness, n (%) | 6 (22.2%) | 8 (28.6%) | 14 (25.5%) |
| Feeling flushed, n (%) | 8 (29.6%) | 5 (17.9%) | 13 (23.6%) |
| Sore eyes, n (%) | 9 (33.3%) | 4 (14.3%) | 13 (23.6%) |
| Upset stomach, n (%) | 5 (18.5%) | 7 (25.0%) | 12 (21.8%) |
| Dizziness, n (%) | 5 (18.5%) | 6 (21.4%) | 11 (20.0%) |
| Sore throat, n (%) | 6 (22.2%) | 3 (10.7%) | 9 (16.4%) |
| Fast heart rate, n (%) | 5 (18.5%) | 2 (7.1%) | 7 (12.7%) |
| Impotence (males only), n (%) | 4/13 (30.8%) | 3/18 (16.7%) | 7/31 (22.6%) |
| *Missing, n (%)* | *0* | *1/18 (5.6%)* | *1/31 (3.2%)* |
| Rash, n (%) | 2 (7.4%) | 3 (10.7%) | 5 (9.1%) |
| Nausea, n (%) | 3 (11.1%) | 2 (7.1%) | 5 (9.1%) |
| Weight loss, n (%) | 2 (7.4%) | 0 | 2 (3.6%) |
| Other, n (%) | 6 (22.2%) | 0 | 6 (10.9%) |
| **EQ-5D-5L** |  |  |  |
| Mean (SD) | 0.8 (0.2) | 0.8 (0.2) | 0.8 (0.2) |
| [Range] | [0.5 to 1.0] | [0.0 to 1.0] | [0.0 to 1.0] |
| *Missing, n (%)* | *0* | *0* | *0* |

Table S3 mean blood pressure measurements from routine clinical notes, pre-randomisation and post-randomisation, including only participants who had both pre- and post-randomisation blood pressure measurements

|  | **Pre-randomisation Intervention (N=20)** | **Post-randomisation Intervention (N=20)** | **Pre-randomisation Control (N=19)** | **Post-randomisation Control (N=19)** |
| --- | --- | --- | --- | --- |
| **Systolic blood pressure (mmHg)** |  |  |  |  |
| Mean (SD) | 142.7 (12.3) | 130.7 (9.9) | 144.6 (9.8) | 134.0 (9.9) |
| Median (IQR) | 140.0 (137.0 to 150.8) | 130.7 (123.3 to 136.3) | 144.3 (138.0 to 149.0) | 133.0 (129.0 to 140.0) |
| [Range] | [116.2 to 172.3] | [111.0 to 151.0] | [129.0 to 170.0] | [110.0 to 152.5] |
| *Missing, n (%)* | *7 (25.9%)* | *7 (25.9%)* | *9 (32.1%)* | *9 (32.1%)* |
| **Diastolic blood pressure (mmHg)** |  |  |  |  |
| Mean (SD) | 78.2 (9.1) | 75.0 (8.6) | 79.2 (6.3) | 73.2 (6.1) |
| Median (IQR) | 80.5 (74.2 to 84.0) | 74.6 (69.6 to 80.8) | 78.7 (74.7 to 85.0) | 72.0 (68.0 to 78.3) |
| [Range] | [60.3 to 90.8] | [60.0 to 95.0] | [68.1 to 92.0] | [62.0 to 84.0] |
| *Missing, n (%)* | *7 (25.9%)* | *7 (25.9%)* | *9 (32.1%)* | *9 (32.1%)* |

Table s4 Summary statistics for defined daily dose of antihypertensive medications at baseline by randomised group

|  | **Intervention (N=27)** | **Control (N=28)** | **Overall (N=55)** |
| --- | --- | --- | --- |
| **Overall Defined Daily Dose** |  |  |  |
| Mean (SD) | 1.9 (3.2) | 1.5 (1.3) | 1.7 (2.4) |
| Median (IQR) | 1.0 (0.0 to 2.0) | 1.0 (0.5 to 2.0) | 1.0 (0.3 to 2.0) |
| [Range] | [0.0 to 15.3] | [0.0 to 6.0] | [0.0 to 15.3] |
| *Missing, n (%)* | *-* | *-* | *-* |
| **Defined Daily Dose ACE inhibitors** |  |  |  |
| Mean (SD) | 0.4 (1.1) | 0.2 (0.8) | 0.3 (1.0) |
| Median (IQR) | 0.0 (0.0 to 0.0) | 0.0 (0.0 to 0.0) | 0.0 (0.0 to 0.0) |
| [Range] | [0.0 to 4.0] | [0.0 to 4.0] | [0.0 to 4.0] |
| *Missing, n (%)* | *-* | *-* | *-* |
| **Defined Daily Dose Alpha 1 Blockers** |  |  |  |
| Mean (SD) | 0.0 (0.1) | 0.0 (0.1) | 0.0 (0.1) |
| Median (IQR) | 0.0 (0.0 to 0.0) | 0.0 (0.0 to 0.0) | 0.0 (0.0 to 0.0) |
| [Range] | [0.0 to 0.8] | [0.0 to 0.5] | [0.0 to 0.8] |
| *Missing, n (%)* | *-* | *-* | *-* |
| **Defined Daily Dose Angiotensin II blockers** |  |  |  |
| Mean (SD) | 0.2 (0.5) | 0.6 (0.9) | 0.4 (0.7) |
| Median (IQR) | 0.0 (0.0 to 0.0) | 0.0 (0.0 to 1.3) | 0.0 (0.0 to 0.5) |
| [Range] | [0.0 to 2.0] | [0.0 to 2.5] | [0.0 to 2.5] |
| *Missing, n (%)* | *-* | *-* | *-* |
| **Defined Daily Dose Beta blockers** |  |  |  |
| Mean (SD) | 0.0 (0.1) | 0.0 (0.1) | 0.0 (0.1) |
| Median (IQR) | 0.0 (0.0 to 0.0) | 0.0 (0.0 to 0.0) | 0.0 (0.0 to 0.0) |
| [Range] | [0.0 to 0.5] | [0.0 to 0.3] | [0.0 to 0.5] |
| *Missing, n (%)* | *-* | *-* | *-* |
| **Defined Daily Dose Calcium Antagonists** |  |  |  |
| Mean (SD) | 1.1 (3.0) | 0.6 (0.7) | 0.8 (2.2) |
| Median (IQR) | 0.0 (0.0 to 1.0) | 0.0 (0.0 to 1.0) | 0.0 (0.0 to 1.0) |
| [Range] | [0.0 to 15.0] | [0.0 to 2.8] | [0.0 to 15.0] |
| *Missing, n (%)* | *-* | *-* | *-* |
| **Defined Daily Dose Centrally acting anti-hypertensives** |  |  |  |
| Mean (SD) | 0.0 (0.0) | 0.0 (0.0) | 0.0 (0.0) |
| Median (IQR) | 0.0 (0.0 to 0.0) | 0.0 (0.0 to 0.0) | 0.0 (0.0 to 0.0) |
| [Range] | [0.0 to 0.2] | [0.0 to 0.0] | [0.0 to 0.2] |
| *Missing, n (%)* | *-* | *-* | *-* |
| **Defined Daily Dose Potassium sparing diuretics** |  |  |  |
| Mean (SD) | 0.0 (0.0) | 0.0 (0.1) | 0.0 (0.1) |
| Median (IQR) | 0.0 (0.0 to 0.0) | 0.0 (0.0 to 0.0) | 0.0 (0.0 to 0.0) |
| [Range] | [0.0 to 0.0] | [0.0 to 0.5] | [0.0 to 0.5] |
| *Missing, n (%)* | *-* | *-* | *-* |
| **Defined Daily Dose Thiazide & related Diuretics** |  |  |  |
| Mean (SD) | 0.1 (0.3) | 0.0 (0.2) | 0.1 (0.3) |
| Median (IQR) | 0.0 (0.0 to 0.0) | 0.0 (0.0 to 0.0) | 0.0 (0.0 to 0.0) |
| [Range] | [0.0 to 1.0] | [0.0 to 1.0] | [0.0 to 1.0] |
| *Missing, n (%)* | *-* | *-* | *-* |

Table S5 Hypertension related consultations from routine clinical notes, pre-randomisation and post-randomisation*

|  | **Pre-randomisation Intervention (N=27)** | **Post-randomisation Intervention (N=27)** | **Pre-randomisation Control (N=28)** | **Post-randomisation Control (N=28)** |
| --- | --- | --- | --- | --- |
| **Any hypertension-related consultation, n(%)** | 16 (59.3%) | 26 (96.3%) | 15 (53.6%) | 28 (100.0%) |
| Mean no. of any hypertension-related consultations (SD) | 1.8 (2.4) | 4.1 (3.5) | 1.6 (2.4) | 1.8 (1.5) |
| Median (IQR) | 1.0 (0.0 to 3.0) | 3.0 (2.0 to 5.0) | 1.0 (0.0 to 2.0) | 1.0 (1.0 to 2.0) |
| [Range] | [0.0 to 10.0] | [0.0 to 13.0] | [0.0 to 9.0] | [1.0 to 8.0] |
| **Any Hypertension-related consultation with GP, n(%)** | 13 (48.1%) | 26 (96.3%) | 12 (42.9%) | 28 (100.0%) |
| **Any hypertension-related consultation with nurse, n(%)** | 8 (29.6%) | 2 (7.4%) | 7 (25.0%) | 2 (7.1%) |

***** The data presented here are pre-randomisation clinical data from 1 year prior to randomisation and post-randomisation clinical data for 1 year after randomisation or up to and including 01/12/20 (whichever came first).

**Table S6 Serious Adverse Events**

| **SAE description** | **Start date of SAE** | **Stop date of SAE** | **Severity of event** | **Reason event classified as serious** | **Outcome of SAE** | **Event related to intervention?** | **Allocation** |
| --- | --- | --- | --- | --- | --- | --- | --- |
| Fractured neck of femur 9 days after randomisation prior to any trial-related intervention. | 22/02/2020 | 28/08/2020 | Severe | life-threatening | Resolved | Not related | Intervention |
| Myocardial infarction with stenting. | 27/04/2020 | 29/04/2020 | Severe | Requiring/prolonging hospitalisation | Resolved | Not related | Intervention |
| Pure Lacunar Infarct following 24h history of R arm and leg weakness. | 11/08/2020 | 12/08/2020 | Moderate | Otherwise significant medical event | Resolved | Not related | Intervention |
